# Rapid Changes in Synaptic Strength After Mild Traumatic Brain Injury

**DOI:** 10.3389/fncel.2019.00166

**Published:** 2019-04-26

**Authors:** Ellen D. Witkowski, Yuan Gao, Alexander F. Gavsyuk, Ido Maor, Gloria J. DeWalt, William D. Eldred, Adi Mizrahi, Ian G. Davison

**Affiliations:** ^1^Department of Biology, Boston University, Boston, MA, United States; ^2^Department of Neurobiology, Edmond & Lily Safra Center for Brain Sciences, Hebrew University of Jerusalem, Jerusalem, Israel

**Keywords:** traumatic brain injury, piriform cortex, synapse, excitatory-inhibitory balance, neuroinflammation

## Abstract

Traumatic brain injury (TBI) affects millions of Americans annually, but effective treatments remain inadequate due to our poor understanding of how injury impacts neural function. Data are particularly limited for mild, closed-skull TBI, which forms the majority of human cases, and for acute injury phases, when trauma effects and compensatory responses appear highly dynamic. Here we use a mouse model of mild TBI to characterize injury-induced synaptic dysfunction, and examine its progression over the hours to days after trauma. Mild injury consistently caused both locomotor deficits and localized neuroinflammation in piriform and entorhinal cortices, along with reduced olfactory discrimination ability. Using whole-cell recordings to characterize synaptic input onto piriform pyramidal neurons, we found moderate effects on excitatory or inhibitory synaptic function at 48 h after TBI and robust increase in excitatory inputs in slices prepared 1 h after injury. Excitatory increases predominated over inhibitory effects, suggesting that loss of excitatory-inhibitory balance is a common feature of both mild and severe TBI. Our data indicate that mild injury drives rapidly evolving alterations in neural function in the hours following injury, highlighting the need to better characterize the interplay between the primary trauma responses and compensatory effects during this early time period.

## Introduction

An estimated 2.8 million Americans suffer a traumatic brain injury (TBI) every year (Taylor et al., 2017), leading to debilitating symptoms such as seizures, cognitive impairment, emotional instability, and sensory deficits (Rimel et al., 1981; Levin et al., 1987, 1990; Lew et al., 2007b; McKee et al., 2009). Mild injuries are most common, accounting for ∼80% of cases (Cassidy et al., 2004). Although mild TBI causes little to no gross damage to neural tissue, its effects often persist for months or even years, significantly impacting employment and quality of life (Rimel et al., 1981; Carroll et al., 2004). Repeated injury significantly worsens both pathology as well as cognitive and motor impairments (Laurer et al., 2001; DeFord et al., 2002; Mouzon et al., 2012). Despite the high prevalence of mild TBI, treatment options remain limited due to our poor understanding of how injury affects neural function, especially in the acute phase after trauma.

Substantial evidence points to deficits in both structural and functional connectivity after mild TBI. Diffuse axonal injury is common, particularly in long-range projections, suggesting potential disruptions in network connectivity (Pandit et al., 2013; Caeyenberghs et al., 2014). TBI is also strongly linked to loss of excitatory-inhibitory (E-I) balance, acute seizures and post-traumatic epilepsy in both humans and animal models, and reduced thresholds for pharmacologically induced seizures in rodents (Lee and Lui, 1992; Nilsson et al., 1994; Annegers et al., 1998; Chadwick, 2000; Golarai et al., 2001; Zanier et al., 2003; Kharatishvili et al., 2006). While overt seizures are less common in mild injury, E-I imbalances have also been described. The hippocampus has shows increased excitability and spontaneous firing within 24 h of mild TBI *in vitro*, and signs of reduced inhibition at 48 h *in vivo* (Reeves et al., 1997; Griesemer and Mautes, 2007). However, other studies found opposing effects, with early increases in inhibition onto CA1 pyramidal cells and reduced excitatory efficacy 1 week later (Santhakumar et al., 2000; Witgen et al., 2005). While cortical effects are less well characterized, existing data also suggest disparate and time-varying effects on E-I balance. Activity in somatosensory cortex was suppressed within 24 h after injury *in vivo* (Alves et al., 2005; Johnstone et al., 2013), but *in vitro* data showed increased excitatory input and action potential firing from 48 h to 1 week post-TBI, and epileptiform activity 1–4 weeks later (Yang et al., 2010; Greer et al., 2012).

While some discrepancies may arise from differences in injury model or experimental system, the complex temporal dynamics of injury responses could also be a key factor. Ding et al. (2011) showed that cortical activity underwent a transition from initial suppression to dramatically elevated excitability at approximately 2 h following injury, indicating that TBI responses can evolve rapidly during this early time window. While TBI effects begin to manifest immediately, few studies have addressed the acute periods spanning the first few hours after injury. Of these, only a handful have made direct intracellular measurements of cellular physiology or synaptic function (Griesemer and Mautes, 2007), so that the neural mechanisms underlying dynamic changes in cortical responsiveness remain open.

Here we examined how mild TBI impacts both excitatory and inhibitory synaptic interactions in cortical circuits, including both a commonly used 48 h time point, but also testing at 1 h post-injury to probe acute injury stages. Using a modified weight drop model allowing free head movement typical of human injury, we consistently found localized neuroinflammatory responses in piriform cortex and neighboring entorhinal areas. To test for associated functional changes, we used whole-cell recordings in acute brain slices to examine synaptic input onto piriform pyramidal neurons at 1 and 48 h after injury. Synaptic function was largely normal at 48 h, but excitatory inputs were substantially increased at 1 h post-TBI. Our data indicate that mild injury causes rapid disruptions in a cortical region that has received little attention, emphasizing the need to consider dynamic effects during immediate post-TBI periods that will be vital to identifying interventions matched to different phases of the injury response.

## Materials and Methods

### Mice

All experiments were performed in adult male and female C57BL/6J mice 2–5 months of age. Animals were group housed in Boston University’s animal care facility on a 12 h light/dark cycle with *ad libitum* access to food and water. All procedures were performed in accordance with the Boston University Institutional Animal Care and Use Committee.

### Injury Model

Mild TBI was induced with a modified Marmarou model where a weight is dropped onto a metal helmet attached to the animal’s skull (Marmarou et al., 1994) and the animal is unrestrained to allow free movement of the head and body to recapitulate the acceleration and shear forces characteristic of human injury (Meaney and Smith, 2011; Kane et al., 2012). Prior to injury, animals were briefly anesthetized, the scalp over the midline was removed, and a stainless steel cylinder was cemented onto the midline at bregma (4 mm diameter; 4 mm tall; Zap Gel, Pacer; C&B Metabond, Parkell). After 3–7 days, mice were briefly anesthetized with isoflurane, given buprenorphine analgesia (0.125 mg/kg, subcutaneous) and placed on top of perforated foil in a custom-made TBI apparatus. A 150–200 g weight secured to fishing line (Stren high impact, Pure Fishing, Inc.) was dropped 150–240 cm through a guide tube onto the helmet implant, propelling the animal through the foil onto foam padding below (5 cm upholstery foam, Mybecca). The fishing line was set to a length that would prevent the possibility of double-hit injury. Animals were monitored in a cage on a heating pad until sternal. Sham groups received identical treatment including brief anesthesia with isoflurane, analgesia and placement in the injury apparatus, and the only difference between groups was the lack of weight drop impact on the helmet implant.

### Behavioral Testing

We confirmed the efficacy of our injury model by investigating vestibulomotor deficits using a custom rotarod apparatus. Mice were placed on a rod that remained stationary for 10 s and then steadily accelerated from 4 to 40 rpm over 5 min. Performance was quantified as the latency to fall. Naïve mice were tested daily (2 trials, 5 min inter-trial interval) for 5 days before injury, at 1 h following TBI on each injury day, and daily for 1 week afterwards. To assess olfactory acuity, we trained mice to perform a binary discrimination between pairs of odorants using a recently described automated home-cage training system (Maor et al., 2019). Briefly, a nose poke into a sampling port triggered pseudorandom delivery of one of two odors. Following a 400 ms delay to enforce a sensory sampling period, mice indicated their choice either by licking in response to the S+ cue to receive a water reward during the 1.6 s response window, or by withholding licking for the S- cue, after which they were free to initiate another trial. S- licks triggered a 2 s timeout period. While mice were allowed to drink freely, all water intake required correct performance in the discrimination task. Mice typically learned to discriminate monomolecular compounds within 4–6 days. Injury was delivered after animals reached a group average criterion of >80%. Animals recovered on a heating pad for 1 h, and were then returned to the odor testing apparatus within 1.5–2 h after injury.

### Immunohistochemistry

In a separate cohort, we examined microglial activation with immunostaining for Iba1, a common neuroinflammatory marker for microglia and macrophages, at time points immediately after two injuries and 24 h after three injuries. Mice were heavily anesthetized with isoflurane and transcardially perfused with ice-cold artificial cerebral spinal fluid (ACSF) followed by 4% paraformaldehyde in 0.1 M phosphate buffer (PB). Brains were harvested and post-fixed overnight in 4% paraformaldehyde in 0.1 M PB at 4°C before undergoing a sucrose series and cryosectioning at 40 μm. Sections were blocked with 5% normal donkey serum in 0.1 M PB with 0.3% Triton X (PBTx) for 1 h at 25°C then incubated overnight in anti-Iba1 rabbit polyclonal antibody 019-19741 (1:1000; Wako Chemicals, United States) diluted in PBTx at 4°C. The primary antibody was visualized by incubating with Alexa Fluor 488 donkey anti-rabbit secondary (1:500; Jackson ImmunoResearch Laboratories, West Grove, PA, United States) diluted in PBTx for 2 h at 25°C. Sections were imaged on a FluoView 300 confocal microscope (Olympus, Center Valley, PA, United States) using a 20× 0.5NA objective and analyzed with ImageJ (NIH, Bethesda, MD, United States). We quantified the total number of Iba1-positive microglia in four different brain areas (somatosensory, entorhinal, and piriform cortex, as well as hippocampus). We also counted the number of visual identified cells with anatomical characteristics associated with activation, such as swollen somata, and calculated a normalized density based on the total measured area in each brain area.

### Electrophysiology

Animals were deeply anesthetized with ketamine/xylazine and transcardially perfused with ice-cold slicing solution containing, in mM: 124 NaCl, 3 KCl, 1.25 NaH_2_PO_4_, 26 NaHCO_3_, 75 sucrose, 10 glucose, 1.3 ascorbic acid, 0.5 CaCl_2_, and 7 MgCl_2_. Acute coronal slices (300 μm) of piriform cortex were cut with a vibratome (VT1200S, Leica, Buffalo Grove, IL, United States) using the same solution, and then transferred to ACSF containing, in mM: 124 NaCl, 3 KCl, 1.25 NaH_2_PO_4_, 26 NaHCO_3_, 20 sucrose, 2 CaCl_2_, and 1.5 MgCl_2_. Both slicing and recordings solutions were continuously oxygenated with 95/5% O_2_/CO_2_. Slices were maintained at 29°C for 30 min for recovery, and then slowly returned to room temperature (approximately 22°C) for incubation and recording. For recording, pyramidal neurons were visualized with a two-photon microscope (Ultima, Prairie Technologies, Middleton, WI, United States) using a 40× 0.8NA objective and Dodt contrast imaging. Whole cell voltage clamp recordings were made with electrodes (3–7 MΩ tip resistance) filled with internal solution containing, in mM: 115 CsMeSO4, 10 HEPES buffer, 10 phosphocreatine disodium, 5 QX314-Cl, 4 MgATP, 0.3 NaGTP, and 0.2 EGTA. Recorded cells were visualized with Alexa 594 to confirm cell type. Membrane voltage was not corrected for liquid junction potential. Spontaneous and evoked excitatory and inhibitory postsynaptic currents (EPSCs and IPSCs) were recorded from superficial pyramidal cells with a Multiclamp 700B amplifier (Molecular Devices, Sunnyvale, CA, United States) and digitized at 10 kHz (National Instruments PCI-6321) using custom Matlab routines (Mathworks, Natick, MA, United States). Evoked synaptic responses were elicited by stimulating the lateral olfactory tract with glass microelectrodes (1–3 MΩ tip resistance) filled with ACSF using a stimulation isolator (World Precision Instruments, Sarasota, FL, United States).

### Data Analysis

Animals were pseudo-randomly assigned to sham or TBI groups. Spontaneous synaptic currents were analyzed in Igor Pro (WaveMetrics, Oregon) using Taro Tools^1^. Raw traces were smoothed using a 3rd order Savitzky-Golay with a 25-point moving window. Event detection thresholds were set between 4 and 6 pA based on noise levels in each recording, and events were inspected visually in each recording to ensure sensitive detection and avoid false positives. The mean thresholds used for experimental comparisons between sham and injury groups were statistically equivalent and varied by <0.3 pA in all cases. Any slow drift in baseline holding current during the recording was removed using a cubic polynomial fit to ensure thresholds were applied consistently to all events. For evoked EPSCs and IPSCs, we calculated the time to peak, width at half maximum amplitude, and exponential decay constants using custom Matlab routines. Decay was best fit with a double exponential of the form *I* = *A* ^∗^ (*e*^-T1^∗^t^ + *e*^-T2^∗^t^), where *A* is maximum amplitude of the current *I*, *t* is time, and T1 and T2 are fast and slow decay constants.

Values from pooled data are reported as mean ± SEM. Statistical significance for group means was calculated using 2-sample *t*-tests, or using 2-sample Kolmogorov–Smirnov tests (*K*–*S*) comparing distributions of event amplitude and frequency, as noted in the text. *K*–*S* tests were performed on group-averaged distributions, calculated individually for each neuron and averaged over all cells in each experimental group. The density of activated microglia across brain areas was analyzed with ANOVA. For *t*-tests, a *p*-value of <0.05 was considered statistically significant. For more sensitive *K*–*S* tests, *p* < 0.01 was considered significant.

## Results

### Mild Injury Induces Behavioral Deficits and Neuroinflammation

**FIGURE 1:**
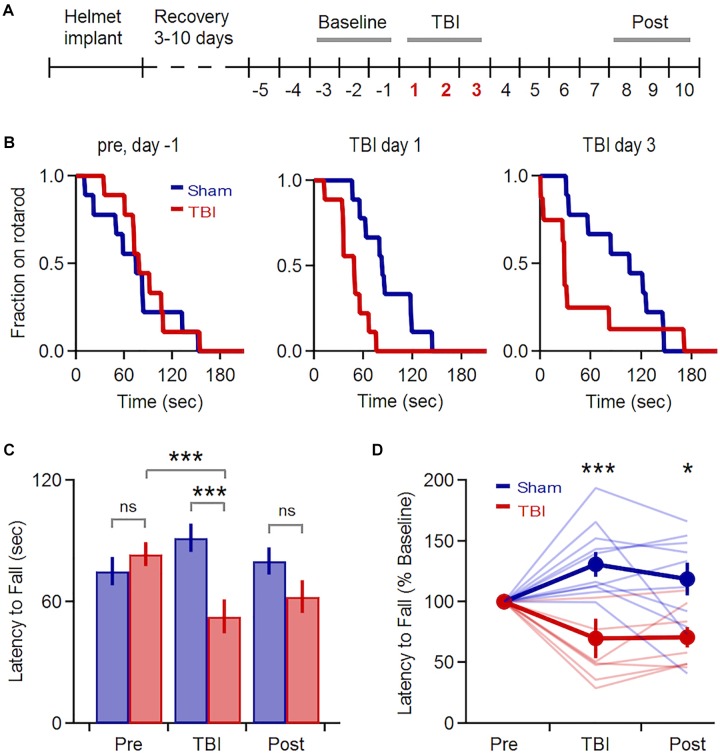
Transient Vestibulomotor Deficits after TBI. **(A)** Timeline of locomotor testing. Latency to fall was measured daily as indicated by numbers. **(B)** Fraction of mice remaining on the rotarod over time for testing 1 day before injury, as well as TBI days 1 and 3. Performance was similar for sham and TBI groups before injury, but was reduced on injury days (day 1: *p* = 0.019; *k* = 0.667; day 3: *p* = 0.034, *k* = 0.639; 2-sample *K*–*S* test; *n* = 9 sham, 8 TBI mice). **(C)** Mean latency to fall averaged over 3-day windows before, during, and after injury. Performance was decreased in TBI mice relative to pre-injury levels [85 ± 11 vs. 55 ± 13 s for baseline and injury days; *p* = 0.0002, *t*(23) = 4.394, paired *t*-test; *n* = 8 mice] and relative to the sham group during the injury period [92 ± 11 vs. 55 ± 13 s for sham and TBI; *p* = 0.002; *t*(49) = –3.242; 2-sample *t*-test; *n* = 9 sham, 8 TBI mice]. **(D)** Group data normalized to pre-injury baseline [injury phase: 131 ± 10% vs. 70 ± 16% for sham and TBI; *p* = 0.005; *t*(15) = 3.321; recovery phase: 118 ± 14% vs. 71 ± 9% for sham and TBI; *p* = 0.012; *t*(15) = 2.866; 2-sample *t*-test; *n* = 9 sham, 8 TBI mice]. ^∗^ and ^∗∗∗^ indicate *p* < 0.05 and *p* < 0.01.

We induced mild TBI using an unrestrained weight-drop model where free head movement recapitulates the acceleration and shear forces characteristic of human injury (Meaney and Smith, 2011; Kane et al., 2012). We began with a multiple-injury paradigm, delivering three repeated impacts at 24 h intervals (150 g, 150–190 cm), mimicking the repetitive TBI that is most strongly associated with human neuropathology (McKee et al., 2009). We first confirmed that our model induced behavioral deficits and neuropathology consistent with mild TBI effects in previous studies (Hamm et al., 1994; Mouzon et al., 2012; Yang et al., 2013; Donat et al., 2017). We assessed vestibulomotor performance using a rotarod assay, starting 5 days before the injury series and continuing for 1 week afterwards (Figure 1A). While pre-injury performance was similar in both groups, TBI significantly reduced latency to fall relative to sham mice over the three injury days (Figure 1B,C: 92 ± 12 vs. 55 ± 13 s for sham and TBI; *p* = 0.002; *n* = 9 sham, 8 TBI mice). Within the TBI group, injury also significantly reduced latency to fall relative to initial baseline levels (85 ± 11 vs. 55 ± 13 s for 3 baseline and injury days; *p* = 0.0002; *n* = 8). Performance in the sham group improved slightly over the same time period, likely from a practice effect (75 ± 11 vs. 92 ± 12 s, for 3 baseline and injury days; *p* = 0.007; *n* = 9 mice). Deficits were also apparent after normalizing each animal’s performance to pre-injury levels (Figure 1D: 131 ± 10 vs. 70 ± 16% for sham and TBI groups averaged over 3 injury days; *p* = 0.005; *n* = 9 sham, 8 TBI mice). The greatest deficits occurred on the 1st and 3rd days of injury (Figure 1B) (*p* = 0.019 and *p* = 0.034 for the 1st and 3rd day of injury; *n* = 9 sham, 8 TBI mice). While performance of the TBI group recovered steadily to near-baseline levels over the following week, it was still reduced relative to sham animals at days 8–10 (Figure 1D: 118 ± 14% vs. 71 ± 9% of pre-injury levels for sham and TBI; *p* = 0.012; *n* = 9 sham, 8 TBI mice). This partial recovery is consistent with other mild TBI studies (Koliatsos et al., 2011; Yang et al., 2013). Overall, we found that injury induces a reliable and relatively long-lasting motor deficit consistent with mild TBI.

**FIGURE 2:**
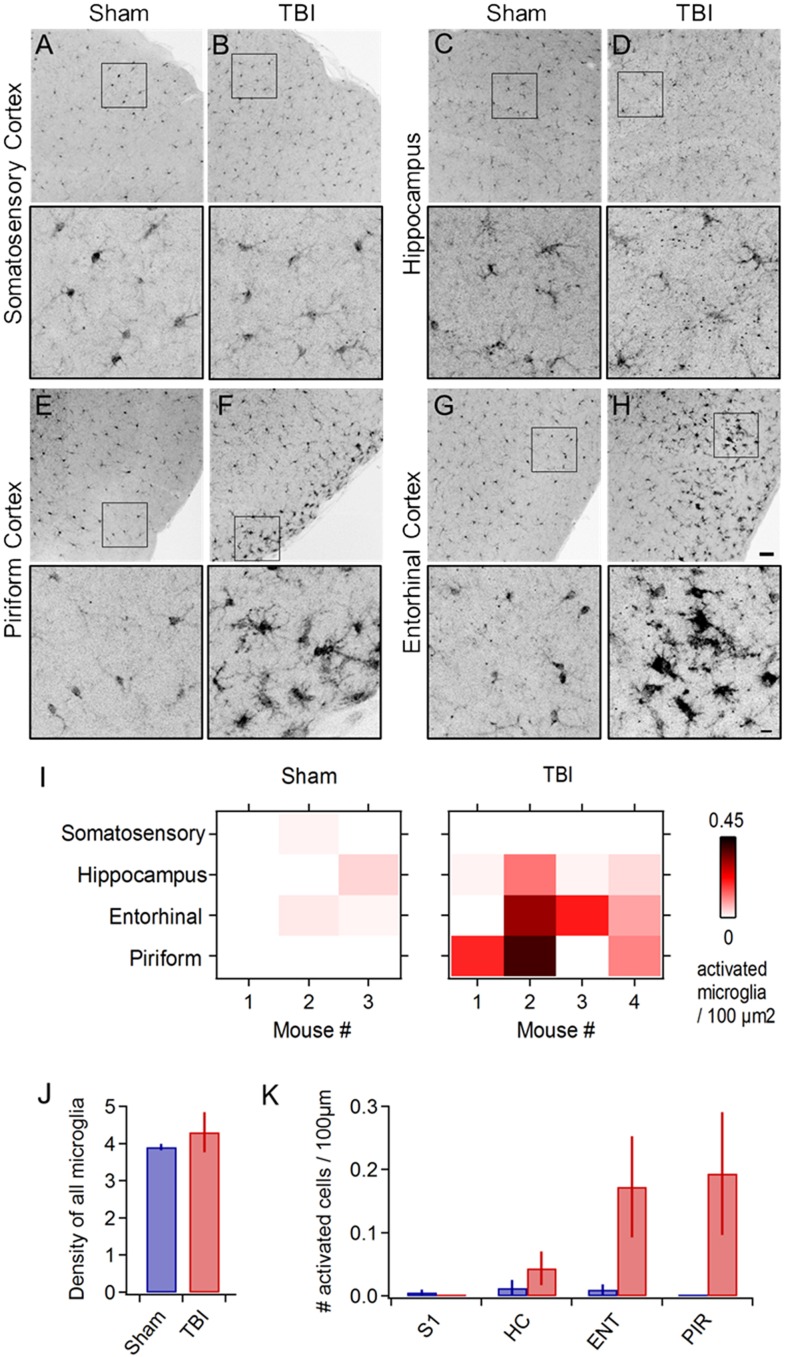
Mild injury leads to microglial activation in lateral inferior temporal lobes. **(A–H)** Iba1 immunostaining reveals microglial morphology in diverse brain regions at 24 h following repeated mild injury. In most regions, microglia appeared largely similar between sham and TBI mice **(A–D)**, but piriform **(E,F)** and entorhinal areas **(G,H)** consistently showed clusters with a swollen appearance characteristic of neuroinflammatory response. Microglia with activated morphology were only infrequently found in sham mice. Scale bars, 50 and 10 μm. **(I)** Heat maps comparing microglial activation across brain areas in individual animals. **(J)** Injury did not alter total microglial density, including both activated and non-activated cells [4.252 ± 0.279 vs. 4.575 ± 0.394 microglia/100 μm^2^ for sham and TBI; *p* = 0.529; *t*(6) = –0.668; 2 sample *t*-test; *n* = 3 sham, 4 TBI mice]. **(K)** Piriform and entorhinal areas showed the largest increases in microglial activation (piriform: 0 ± 0 vs. 0.194 ± 0.097 activated microglia/100 μm^2^ for sham and TBI; entorhinal: 0.010 ± 0.008 vs. 0.173 ± 0.080 activated microglia/100 μm^2^ for sham and TBI).

In addition to behavioral disruptions, TBI commonly leads to neuropathology including pro-inflammatory responses in astrocytes and microglia, which develop a swollen ‘activated’ morphology, migrate to the injury site, and release inflammatory cytokines (Kreutzberg, 1996; Streit, 2000; Nimmerjahn et al., 2005; Loane and Byrnes, 2010). Microglial activation is reliably seen across diverse TBI models (Kaur et al., 1995; Davalos et al., 2005; Shitaka et al., 2011; Goldstein et al., 2012; Xu et al., 2016; Tagge et al., 2018). To probe neuroinflammatory responses, we used Iba1 immunohistochemistry to label microglia and macrophages (Imai et al., 1996; Ito et al., 1998) and examined their morphology across a wide range of brain areas including neocortex, hippocampus, striatum, and piriform and entorhinal cortices. While microglia in sham animals appeared normal (Figure 2A,C,E,G), TBI led to pronounced morphological changes including swollen somata and processes indicative of activation (Figure 2F,H). Neuroinflammation was apparent at 24 h following 3 injuries, and in one case immediately following the second injury (Figure 2: *n* = 3 sham, 4 TBI mice). In each brain region, we quantified the number and density of cells showing anatomical changes characteristic of an activated state (Figure 2I–K). Microglial activation was reliably localized to the lateral inferior temporal lobes, particularly to piriform and entorhinal cortices (Figure 2F,H) and olfactory tubercle (not shown), regions that have received little attention in TBI. Activated cells were often concentrated near the cortical surface (Figure 2F,H). Interestingly, we rarely found activation directly under the injury site near somatosensory cortex or in the hippocampus (Figure 2B,D), which has commonly been seen after more severe injury (Reeves et al., 1995, 1997; Santhakumar et al., 2000, 2001; Witgen et al., 2005), perhaps due to differences in severity, impact site, or head movement in our model. Overall, however, these reliable and localized inflammatory responses are consistent with mild injury.

### Mild TBI Has Moderate Effects on Synaptic Input Onto Pyramidal Cells at 48 h

**FIGURE 3:**
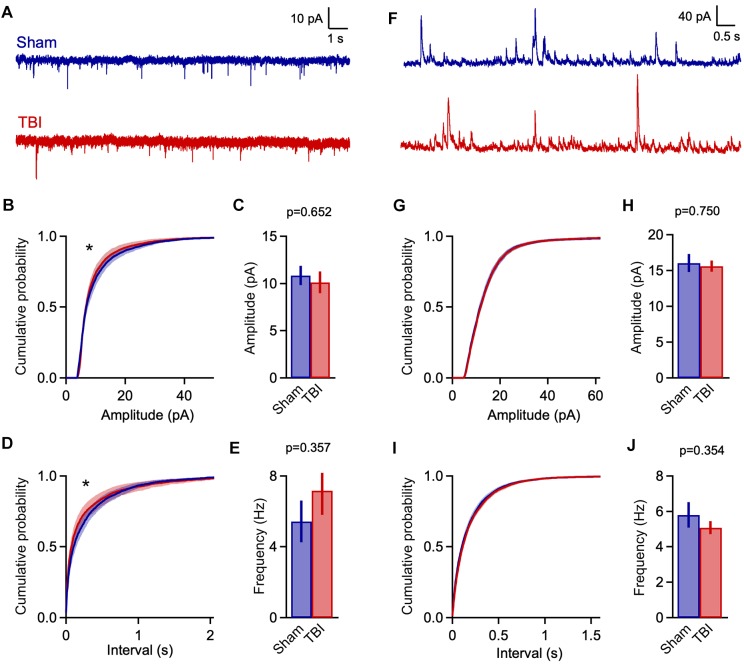
Mild TBI has comparatively minor effects on spontaneous synaptic input 48 h post-injury. **(A)** Whole cell recordings of excitatory input onto superficial pyramidal cells in piriform cortex. **(B,C)** Amplitudes of excitatory events showed moderate changes after TBI. Left, cumulative histograms (*p* = 0.009; *k* = 0.056; *K*–*S* test; *n* = 1750 sham, 1377 TBI events). Right, mean values [10.8 ± 1.0 vs. 10.1 ± 1.2 pA for sham and TBI; *p* = 0.652, *t*(29) = –0.456; 2-sample *t*-test; *n* = 9 mice, 14 cells for sham; 11 mice, 17 cells for TBI]. **(D,E)** Interevent interval and mean frequency for excitatory events. The sEPSC interval distribution shifted slightly toward smaller values after TBI (*p* = 0.0003; *k* = 0.074; *K*–*S* test; *n* = 1750 sham, 1377 TBI events) but mean values were not significantly changed [5.4 ± 1.2 vs. 7.2 ± 1.4 Hz for sham and TBI; *p* = 0.898, 2-sample *t*-test; *t*(29) = 0.129; *n* = 9 mice, 14 cells for sham; 11 mice, 17 cells for TBI]. **(F)** Spontaneous inhibitory inputs onto the same superficial pyramidal cell recorded at excitatory reversal potential. **(G,H)** Cumulative histograms and mean values for sIPSC amplitude, which were unchanged by TBI. **(I,J)** Cumulative histogram of interevent interval and mean frequency values for inhibitory events, which were also unaffected. [*p* = 0.026; *k* = 0.038; *K*–*S* test; *n* = 2744 sham, 3213 TBI events; 5.8 ± 0.7 vs. 5.1 ± 0.4 Hz for sham and TBI; *p* = 0.754; *t*(29) = 0.317; 2 sample *t*-test; *n* = 9 mice, 14 cells for sham; 11 mice, 17 cells for TBI]. ^∗^ indicates *p* < 0.01 in *K*–*S* tests.

Traumatic brain injury-induced deficits may arise at least in part from disrupted synaptic communication. While prior work has shown both increased cellular excitability (Griesemer and Mautes, 2007) and diffuse axonal injury leading to functional disconnection (Pandit et al., 2013), there are few direct measurements of how synaptic input is affected by mild TBI. The consistent presence of neuroinflammation in piriform led us to use this region to probe the functional effects of mild injury. We used whole-cell recordings in acute brain slices to test for TBI-induced changes in synaptic strength, examining both spontaneous and evoked synaptic input onto superficial pyramidal cells, the major excitatory cell type in this brain area (Figure 3).

Despite finding reliable microglial activation in piriform, we observed only moderate changes in spontaneous synaptic currents measured 48 h after our repeated 3-injury mild TBI series. The mean amplitude of excitatory events was not statistically different between sham and TBI groups (Figure 3A,C: 10.8 ± 1.0 vs. 10.1 ± 1.2 pA for sham and TBI; *p* = 0.652; *n* = 9 mice, 14 cells for sham, 9 mice, 17 cells for TBI). Frequency was similarly unchanged (Figure 3E: 5.4 ± 1.2 vs. 7.2 ± 1.4 Hz for sham and TBI; *p* = 0.898; *n* = 9 mice, 14 cells for sham; 9 mice, 17 cells for TBI). While the more sensitive Kolmogorov–Smirnov (*K*–*S*) test did reveal a significant shift in the distributions of both amplitude and interevent intervals (Figure 3B,D: *p* = 0.009 and *p* = 0.0003 for amplitude and interval; *n* = 1750 sham, 1377 TBI events), changes in synaptic function were less pronounced than in other models of moderate-to-severe TBI (Witgen et al., 2005; Cantu et al., 2015). Overall, our data showed significant but comparatively limited effects on excitatory synaptic inputs.

Traumatic brain injury has also been linked to changes in inhibitory function (Reeves et al., 1997; Witgen et al., 2005; Smith et al., 2015), which plays a prominent role in balancing local excitatory networks in piriform (Luna and Schoppa, 2008; Franks et al., 2011; Suzuki and Bekkers, 2011; Sheridan et al., 2014; Large et al., 2016; Bolding and Franks, 2018). Recording from the same pyramidal neurons, we examined inhibitory input by holding at excitatory reversal potential (+5 mV; Figure 3F). Similar to excitatory input, there was little to no change in either mean amplitude or frequency of sIPSCs after injury (Figure 3H,J: amplitude: 16.0 ± 1.2 vs. 15.6 ± 0.8 pA for sham and TBI; *p* = 0.750; frequency: 5.8 ± 0.7 vs. 5.1 ± 0.4 Hz for sham and TBI; *p* = 0.754; *n* = 9 mice, 14 cells for sham; 9 mice, 17 cells for TBI). Again, the higher-sensitivity *K*–*S* test revealed a shift toward increased sIPSC intervals after injury (Figure 3I: *p* = 0.026, *n* = 2744 sham, 3213 TBI events), but this effect was moderate in size and did not reach our statistical criterion. Altogether, while injury altered the distribution of both excitatory and inhibitory inputs between sham and TBI animals, there was little effect on mean amplitude or frequency, suggesting that mild trauma either had a relatively minor impact on synaptic transmission or that compensatory effects were already recruited at 48 h after injury.

Finally, to further probe how injury acts on piriform circuit function, we measured electrically evoked responses upon stimulating afferent axons of the lateral olfactory tract (LOT), which provides sensory input from olfactory bulb. LOT responses contain multiple components reflecting local interactions within piriform, including direct excitatory input from olfactory bulb, additional intracortical excitation from other local pyramidal neurons, and local inhibition from both feedforward and feedback inhibitory neurons (Suzuki and Bekkers, 2007, 2011; Luna and Schoppa, 2008; Franks et al., 2011; Large et al., 2016). Given the importance of E-I balance in cortical function in general (Xue et al., 2014), and in piriform in particular (Bolding and Franks, 2018), we tested how injury affects the relative contributions of excitation and inhibition in piriform pyramidal cells. Similar to spontaneous measurements, we first isolated excitatory inputs by holding at -70 mV. LOT stimulation produced rapid onset, presumably monosynaptic EPSCs, as well as a secondary component presumably due to local recurrent circuits. For consistency, we adjusted stimulus intensity to elicit similar levels of input in each cell (∼200 pA). We then measured corresponding GABAergic currents at the same stimulus intensity while holding at excitatory reversal (+5 mV). Inhibitory currents scaled with excitatory responses but were typically larger, consistent with previous data in both piriform and neocortex and reflecting the higher conductance of GABA_A_ receptors (Franks et al., 2011; Isaacson and Scanziani, 2011; Haider et al., 2012; Xue et al., 2014; Large et al., 2016, 2018).

**FIGURE 4:**
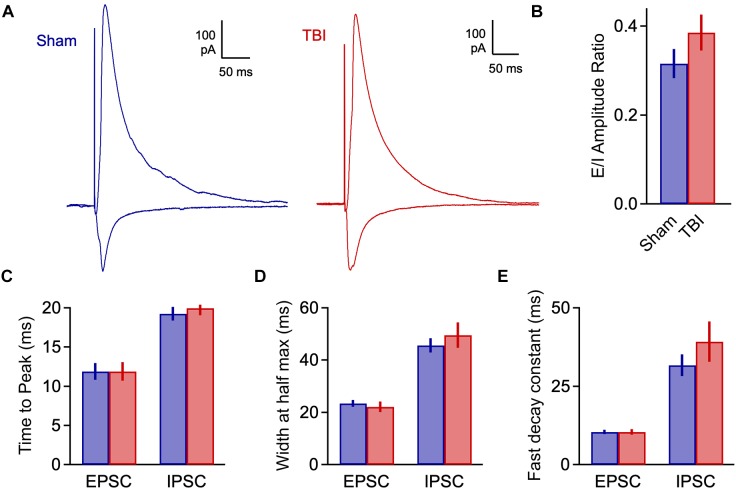
Excitatory-inhibitory balance remains stable at 48 h post-injury. **(A)** Excitatory and inhibitory inputs in the same piriform pyramidal cell upon electrical stimulation of the LOT. **(B)** The ratio of excitatory to inhibitory amplitudes was largely unchanged at 48 h after TBI [0.316 ± 0.033 vs. 0.386 ± 0.040 for sham and TBI; *p* = 0.185; *t*(29) = –1.357; *t*-test; *n* = 9 mice, 16 cells for sham; 9 mice, 15 cells for TBI]. **(C)** Response kinetics (time to peak) were unchanged for both EPSCs and IPSCs [EPSCs: 11.9 ± 1.0 vs. 11.9 ± 1.2 ms for sham and TBI; *p* = 0.630; *t*(26) = 0.488; *t*-test; IPSCs: 19.3 ± 0.9 vs. 20.0 ± 0.9 ms for sham and TBI; *p* = 0.580; *t*(27) = –0.56; *t*-test]. **(D,E)** Duration (width at half max) and decay kinetics were also unchanged [half width – EPSCs: 23.4 ± 1.3 vs. 22.0 ± 2.0 ms for sham and TBI; *p* = 0.294; *t*(26) = 1.07; *t*-test; IPSCs: 45.6 ± 2.8 vs. 49.5 ± 4.9 ms; *p* = 0.491; *t*(27) = –0.699; *t*-test; decay time – EPSCs: 10.4 ± 0.7 vs. 10.4 ± 0.8 ms; *p* = 0.308; *t*(26) = 1.040; *t*-test; IPSCs: 31.6 ± 3.5 vs. 39.2 ± 6.4 ms; *p* = 0.302; *t*(27) = –1.051; *t*-test]. For EPSC kinetics, *n* = 9 mice, 14 cells for sham; 9 mice, 15 cells for TBI; for IPSC kinetics, *n* = 9 mice, 13 cells for sham; 9 mice, 14 cells for TBI.

We quantified an E/I ratio for each cell based on the peak amplitudes of excitatory and inhibitory currents. Consistent with the minor effects on spontaneous input, E/I balance was not significantly altered at 48 h after injury (Figure 4A,B: 0.316 ± 0.033 vs. 0.386 ± 0.040 for sham and TBI; *p* = 0.185; *n* = 9 mice, 16 cells for sham; 9 mice, 15 cells for TBI). We also tested for potential changes in the time course of synaptic responses, which could also affect the interplay between excitation and inhibition independently of peak amplitude. We found no effect on response kinetics as assessed by time to peak, width at half maximum, or fast or slow decay time constants (Figure 4C–E: peak time: 11.9 ± 1.0 vs. 11.9 ± 1.2 ms for eEPSCs, 19.3 ± 0.9 vs. 20.0 ± 0.9 ms for eIPSC; half width: 23.4 ± 1.3 vs. 22.0 ± 2.0 ms for eEPSCs, 45.6 ± 2.8 vs. 49.5 ± 4.9 ms for eIPSCs; fast decay constant: 10.4 ± 0.7 vs. 10.4 ± 0.8 for eEPSCs, 31.6 ± 3.5 vs. 39.2 ± 6.4 ms for eIPSCs). These data further suggest that mild injury either has relatively minor impact on synaptic communication, or that effects have largely recovered at 48 h.

### Mild TBI Drives Rapid Loss of E-I Balance at 1 h Post-Injury

The moderate synaptic changes seen at 48 h post-injury were unexpected given the behavioral and neuroinflammatory effects we observed. TBI responses can evolve rapidly *in vivo* during the hours after injury, including successive periods of reduced and heightened excitability (Kharatishvili et al., 2006; Ding et al., 2011; Hansen et al., 2018). Compensatory effects can also contribute to recovery as early as 48 h (Griesemer and Mautes, 2007; Greer et al., 2012), perhaps accounting for the apparent stability in synaptic input. To address the direct effects of TBI at early post-injury phases independently of longer-term compensatory changes, we instead delivered a single weight drop and measured synaptic activity in slices prepared 1 h after impact. Data for this early injury phase are particularly limited, especially for mild closed-skull TBI (Reeves et al., 2000; Griesemer and Mautes, 2007). Specifically, we probed for changes in synaptic input and E-I balance linked to seizure and post-traumatic epilepsy in human injury (Annegers et al., 1998). While loss of E-I balance has been previously described, the direction and timing of effects has been inconsistent across studies.

**FIGURE 5:**
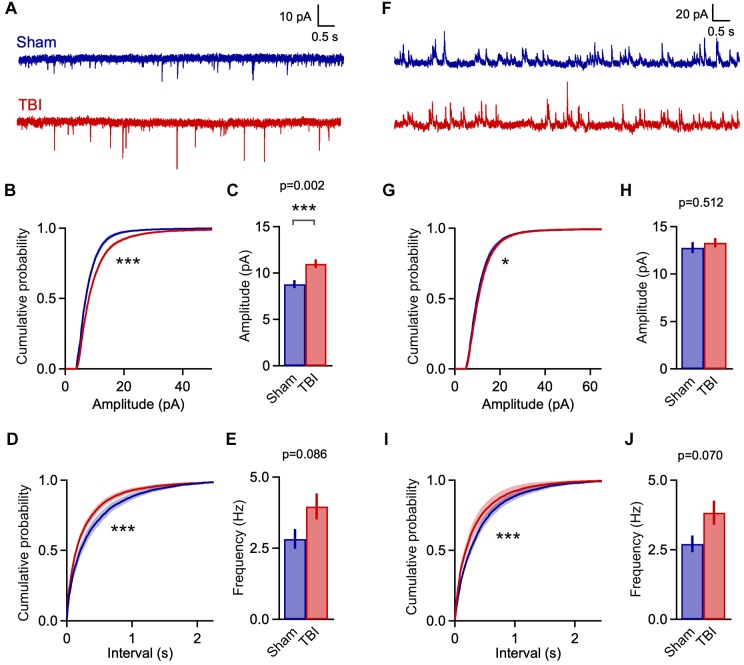
Rapid increases in excitatory input 1 h after mild TBI. **(A)** Example traces showing spontaneous EPSCs at 1 h after a single injury. **(B,C)** Cumulative histograms and mean values for sEPSC amplitudes. Excitatory events were significantly larger in the TBI group [*p* = 1e^-08^, *k* = 0.122; *K*–*S* test; *n* = 2016 sham, 4050 TBI events; 8.8 ± 0.4 vs. 11.0 ± 0.5 pA for sham and TBI; *p* = 0.0025; *t*(46) = 3.201; 2 sample *t*-test; *n* = 8 mice, 18 cells for sham; 12 mice, 30 cells for TBI]. **(D,E)** Cumulative histograms for sEPSC interval and bar plot of mean frequency. TBI also decreased the interval between excitatory inputs (*p* = 1e^-08^, *k* = 0.101; *K*–*S* test; *n* = 2016 sham, 4050 TBI events), although mean frequency showed only a trend toward increase [2.8 ± 0.4 vs. 4.0 ± 0.4 Hz for sham and TBI; *p* = 0.086; *t*(46) = 1.755; 2 sample *t*-test; *n* = 8 mice, 18 cells for sham; 12 mice, 30 cells for TBI]. **(F)** Example traces showing spontaneous inhibitory inputs. **(G,H)** TBI caused a slight shift toward larger amplitudes (*p* = 0.0002, *k* = 0.049; *K*–*S* test; *n* = 2664 sham, 6810 TBI events), although mean sIPSC size was unaffected [12.8 ± 0.6 vs. 13.3 ± 0.5 pA for sham and TBI; *p* = 0.512; *t*(46) = 0.661; 2 sample *t*-test; *n* = 8 mice, 18 cells for sham; 12 mice, 30 cells for TBI]. **(I,J)** TBI altered the distribution toward more frequent inhibitory inputs (*p* = 1e^-08^; *k* = 0.102; *K*–*S* test; *n* = 2664 sham, 6810 TBI events), although mean frequency showed only a trend [2.7 ± 0.3 vs. 3.8 ± 0.4 Hz for sham and TBI; *p* = 0.070; *t*(46) = 1.860; 2 sample *t*-test; *n* = 8 mice, 18 cells for sham; 12 mice, 30 cells for TBI]. ^∗^ and ^∗∗∗^ indicate *p* < 0.05 and *p* < 0.01 for *t*-tests, and *p* < 0.01 and *p* < 0.0001 for *K*–*S* tests.

Despite the use of a single rather than a repetitive injury paradigm, TBI-induced changes in synaptic function were considerably more pronounced at 1 h post-injury than at 48 h after three injuries. TBI significantly shifted the distributions for both sEPSC amplitude and interval toward larger and more frequent events (Figure 5A,B,D: *p* = 1e^-08^ for amplitude; *p* = 1e^-08^ for frequency; *n* = 2016 sham, 4050 TBI events). Mean sEPSC amplitudes were also significantly increased (Figure 5C: 8.8 ± 0.4 vs. 11.0 ± 0.5 pA for sham and TBI; *p* = 0.0025; *n* = 8 mice, 18 cells for sham; 12 mice, 30 cells for TBI). Mean frequency showed a slight but non-significant upward trend as well (Figure 5E: 2.8 ± 0.4 and 4.0 ± 0.4 Hz for sham and TBI; *p* = 0.086, *n* = 8 mice, 18 cells for sham; 12 mice, 30 cells for TBI). These data indicate that mild injury does in fact cause robust effects on excitatory synaptic function, but that these are short-lived compared to more severe models.

Enhanced excitatory input was accompanied by a comparable shift toward shorter sIPSC intervals at 1 h post-injury (Figure 5F,I: *p* = 1e^-08^; *n* = 2664 sham, 6810 TBI events), although mean frequency was not significantly affected (Figure 5J: 2.7 ± 0.3 and 3.8 ± 0.4 Hz for sham and TBI; *p* = 0.07, *n* = 8 mice, 18 cells for sham; 12 mice, 30 cells for TBI). While mean inhibitory amplitude was also unchanged, there was a slight but statistically significant shift in the sIPSC amplitude distribution (Figure 5G,H: *p* = 0.0002, *n* = 2664 sham, 6810 TBI events; 12.8 ± 0.6 vs. 13.3 ± 0.5 pA for sham and TBI; *p* = 0.512; *n* = 8 mice, 18 cells for sham; 12 mice, 30 cells for TBI). Overall, injury drove clear increases in the rate of input onto pyramidal neurons that were comparable for excitatory and inhibitory synapses. The amplitude of excitatory inputs also showed a substantial increase that far outweighed the less robust changes in inhibitory amplitude.

**FIGURE 6:**
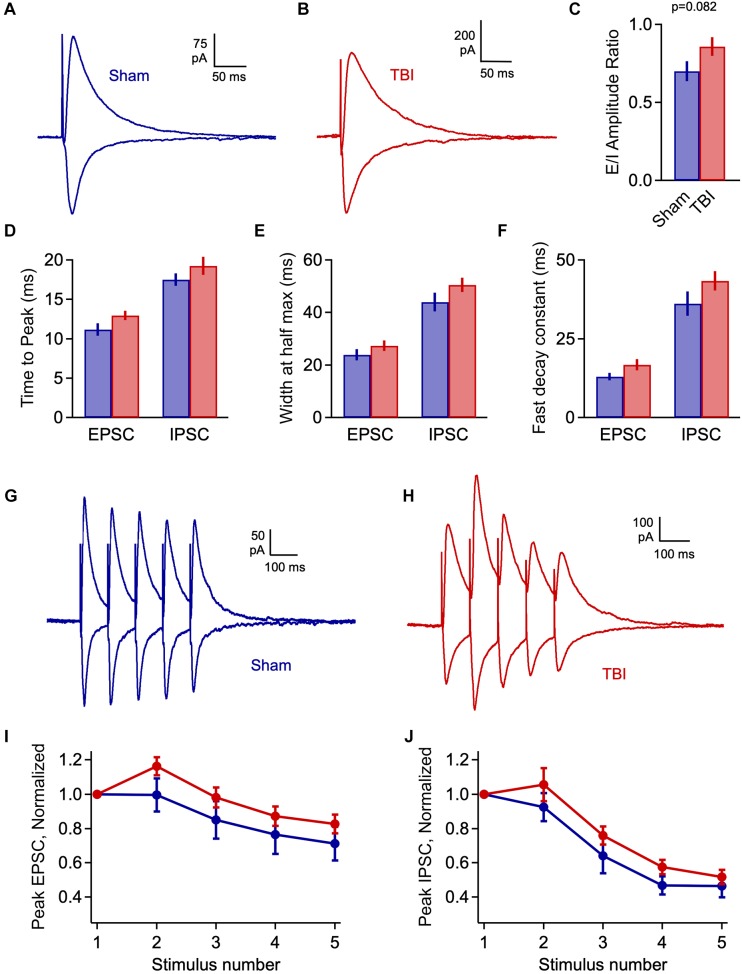
Injury has limited effects on evoked E-I balance 1 h post-injury. **(A,B)** Excitatory and inhibitory inputs in piriform pyramidal cells elicited by LOT stimulation. **(C)** Excitatory-inhibitory ratio was slightly but non-significantly increased after TBI [0.700 ± 0.065 vs. 0.858 ± 0.060 for sham and TBI; *p* = 0.082; *t*(66) = 1.76; 2 sample *t*-test; *n* = 9 mice, 28 cells for sham; 12 mice, 40 cells for TBI]. **(D)** Time to peak is not significantly changed by injury [EPSCs: 11.2 ± 0.8 vs. 12.9 ± 0.6 ms for sham and TBI; *p* = 0.074; *t*(59) = –1822; 2 sample *t*-test; IPSCs: 17.5 ± 0.8 vs. 19.3 ± 1.1 ms; *p* = 0.280; *t*(61) = –1.090; *t*-test]. **(E,F)** Width at half-maximum and decay time constant were not significantly affected [half width – EPSCs: 23.8 ± 2.1 vs. 27.3 ± 2.0 ms; *p* = 0.266; *t*(59) = –1.122; IPSCs: 43.9 ± 3.5 vs. 50.5 ± 2.8 ms; *p* = 0.151; *t*(61) = –1.454; decay time constant: EPSCs: 12.9 ± 1.2 vs. 16.7 ± 1.7 ms; *p* = 0.148; *t*(59) = –1.466; IPSCs: 36.2 ± 3.8 vs. 43.4 ± 3.1 ms; *p* = 0.152; *t*(61) = –1.450]. 2-sample *t*-test used for all kinetics comparisons: EPSCs, *n* = 9 mice, 22 cells and 12 mice, 39 cells for sham and TBI; IPSCs, *n* = 9 mice, 23 cells and 9 mice, 40 cells for sham and TBI. **(G,H)** Example responses to 10-Hz trains of LOT stimulation. **(I,J)** Short-term synaptic plasticity for excitatory (left) and inhibitory (right) responses, which was slightly but not significantly enhanced (EPSCs: *p* > 0.14 for all pulses; IPSCs: *p* = >0.19 for all pulses).

Elevated excitatory input, if not matched by increases in inhibition, could lead to a potential imbalance in these circuit elements during early injury phases. To test this idea, we again measured the excitatory and inhibitory components of evoked LOT responses at 1 h after TBI. E-I ratios showed considerable variability across cells, and while there was a trend toward greater excitation, this shift was non-significant (Figure 6A–C: 0.700 ± 0.065 vs. 0.858 ± 0.060 for sham and TBI; *p* = 0.082; *n* = 9 mice, 28 cells for sham; 12 mice, 40 cells for TBI). There was also no significant change in response kinetics for either EPSCs or IPSCs, including time to peak, width at half maximum, and decay constants (Figure 6D–F: peak time: 11.2 ± 0.8 vs. 12.9 ± 0.6 ms for eEPSCs, 17.5 ± 0.8 vs. 19.3 ± 1.1 ms for eIPSCs; half width: 23.8 ± 2.1 vs. 27.3 ± 2.0 ms for eEPSCs, 43.9 ± 3.5 vs. 50.5 ± 2.8 ms for eIPSCs; fast decay constant: 12.9 ± 1.2 vs. 16.7 ± 1.7 for eEPSCs, 36.2 ± 3.8 vs. 43.4 ± 3.1 ms for eIPSC), although all of these parameters consistently showed slight increases. Finally, we tested for potential changes in the short-term dynamics of synaptic strength that could alter E-I balance and circuit function, by stimulating the LOT with a train of 5 pulses at 10 Hz. Again, the relative amplitude of both excitatory and inhibitory currents was slightly increased relative to sham for all subsequent pulses in the train, but this effect was not significant (Figure 6G–J). In general, injury led to minor effects on LOT-evoked responses that were in a direction consistent with spontaneous data, but were less robust and did not reach statistical significance.

**FIGURE 7:**
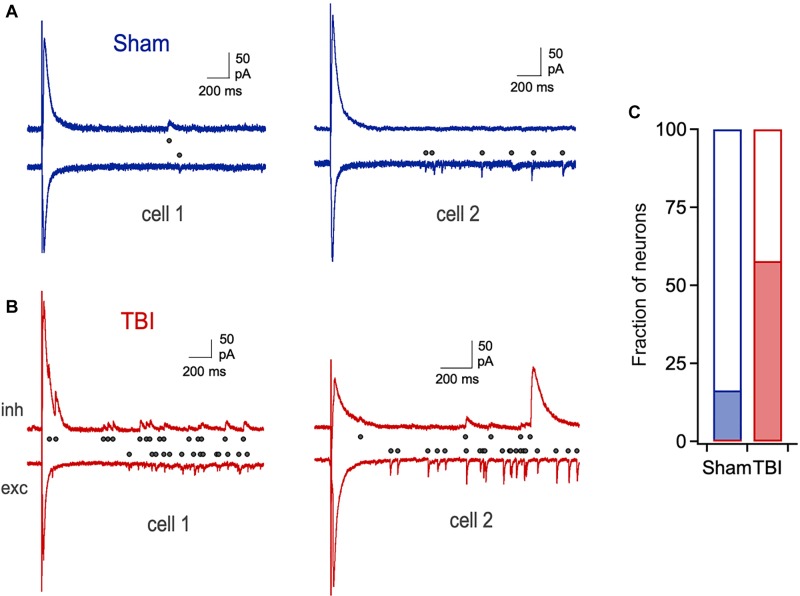
Prolonged, reverberating synaptic responses after mild TBI. **(A)** Typical brief responses to LOT stimulation in sham animals, which showed only rare cases of late synaptic activity (right). Dots indicate individual synaptic currents. **(B)** Examples of unusually long-lasting barrages of synaptic input after injury. **(C)** The prevalence of prolonged activity is greatly increased by TBI (16.0% vs. 56.4% for sham and TBI).

Despite the lack of significant changes in amplitude and kinetics of evoked monosynaptic responses, cells from the TBI group frequently showed unusual extended barrages of synaptic input that could persist for several seconds after the stimulus (Figure 7A,B). While a small fraction of neurons in the sham group displayed a smaller degree of prolonged activity, its prevalence and intensity were strongly increased after TBI (Figure 7C: 16.0% vs. 56.4% of cells from sham and TBI groups, respectively). While the source of this long-lasting reverberating activity is unclear, it suggests a disruption in the normal circuit interactions that typically limit activity in piriform to brief time windows (Luna and Schoppa, 2008; Bolding and Franks, 2018).

In general, relative levels of excitation and inhibition were less affected in evoked than in spontaneous inputs, perhaps due to differences in how these data emphasize afferent versus intracortical inputs. Overall, however, our major finding at 1 h post-injury was a rapid enhancement of excitatory input that appeared to outweigh increases in inhibition, which was paralleled by unusual long-lasting bouts of synaptic input persisting for seconds after stimulation. Together, these data suggest that loss of E-I balance is a common feature of both mild and severe injury.

### Mild Injury Leads to Sensory Deficits in Odor Discrimination

**FIGURE 8:**
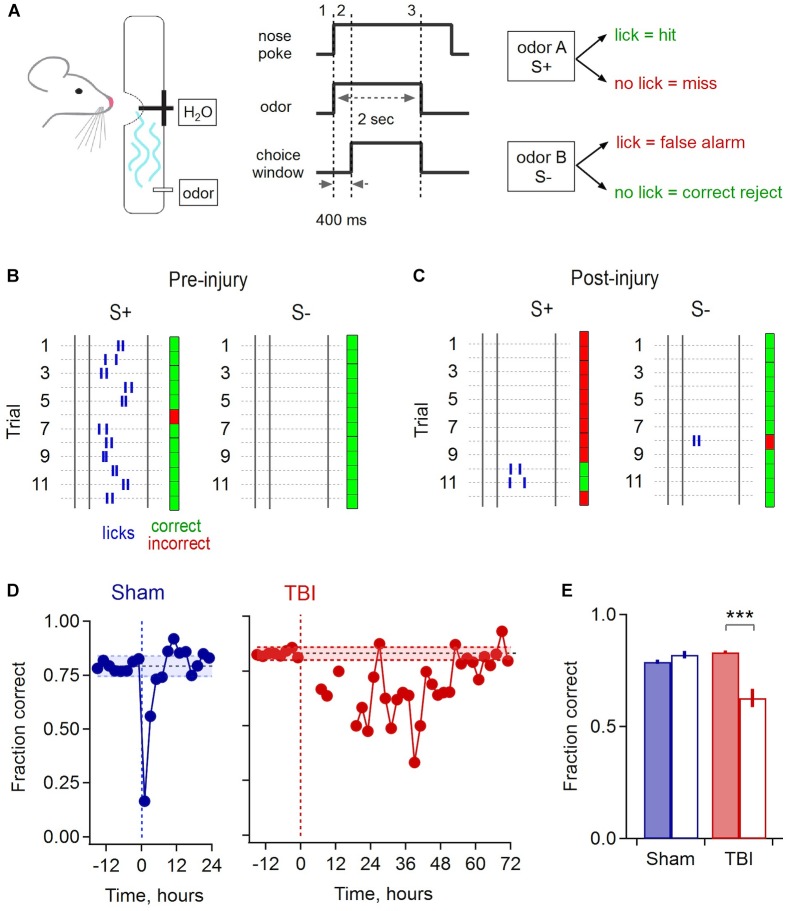
Olfactory deficits after mild injury. **(A)** Go/No-go odor discrimination task. Animals sampled from an odor port containing a lick tube, and after a brief delay reported their choice of odor A (S+, rewarded) vs. odor B (S–, unrewarded). **(B)** Block of 24 example trials for one animal preceding injury, grouped by S+ and S– odors to illustrate accurate performance. Rasters indicate licks, and green and red indicate correct and incorrect choices. The first 2 licks for each trial are shown. **(C)** Example trials for the same animal following injury. **(D)** Group data shows a brief drop in performance due to sham treatment that recovers after ∼4 h (mean accuracy prior to injury, 0.793 ± 0.024 and 0.83 ± 0.014 for sham and TBI; shading shows mean ± 2 SD). Mice performed few trials during the 12–24 h after injury, after which they showed a drop in performance lasting until ∼48 h. **(E)** Pre- and post-injury accuracy for sham and TBI groups. Sham mice had indistinguishable performance during the 12 h pre-injury and the 12–24 h post-recovery period [0.790 ± 0.010 vs. 0.822 ± 0.018, respectively; *p* = 0.145, *t*(10) = –1.58, paired sample *t*-test; *n* = 6 time points pre and post-injury]. TBI mice showed a significantly loss of performance over the 24–48 h post-injury period, the point where they resumed consistent sampling, compared to the 12 h pre-injury [pre, 0.832 ± 0.007 vs. post, 0.628 ± 0.040; *p* = 0.0027; *t*(16) = 3.547; 2 sample *t*-test; *n* = 6 pre and 12 post-injury time points] ^∗∗∗^ indicates *p* < 0.01.

To address the functional correlates of synaptic disruption and inflammatory responses in piriform, we used a behavioral assay to test how sensory acuity was affected by mild injury. We trained mice to discriminate between pairs of odorants in a Go/No-go paradigm, where licking in response to the S+ odor was rewarded by delivery of a water drop, and the second S- odor required animals to withhold licking to avoid receiving a timeout (Figure 8A). We used a recently described automated training system where mice are continuously group housed in their home cage and task performance is monitored throughout using RFID tags (Maor et al., 2019). Animals were allowed to drink freely at all times, but all water intake required correct discrimination. This approach maintained normal physiological status at the time of injury, avoiding potential confounds due to combining TBI with water restriction.

Mice typically learned the initial task structure and began discriminating between odors within ∼4–6 days. Sham and injury treatments were performed after animals reached a mean performance level of 80%, where they reliably licked in response to the rewarded S+ cue and withheld licking to the S- odor (Figure 8B). While there was a brief drop in performance immediately after sham treatment, likely due to stress from removal from the homecage and brief anesthesia, accuracy rapidly recovered to previous levels within approximately 4 h. In contrast, most TBI animals refrained from drinking for up to 12–24 h after mild injury, apart from occasional sporadic trials. Error rates then rose considerably in subsequent trials. Interestingly, initial errors were biased toward ‘miss’ responses, suggesting that mice may not have recognized the target odor, or alternatively could have acquired hypersensitivity (Figure 8C). Performance remained degraded for a period of ∼48 h, far outlasting the effects of sham treatment, before slowly recovering to pre-injury levels (Figure 8D). To quantify the loss of discrimination, we calculated the average group performance for the period from 24 to 48 h after injury, which dropped to 62.8% during this period compared to an average of 83.2% before TBI (Figure 8E). These results indicate that disrupted synaptic communication in piriform is associated with a sensory deficit in odor discrimination that persists for a period of ∼48 h, although effects on other task-related brain areas may contribute as well.

## Discussion

While several studies have characterized changes in neural activity early after TBI, there is a lack of consensus on the direction of E-I shifts, and little work has directly addressed how synaptic interactions are affected acutely after trauma, particularly in mild forms of TBI. Here we characterized synaptic changes in piriform cortex, which showed consistent neuroinflammatory responses in our mild injury model and has been linked to generation and propagation of seizures in other paradigms (Piredda and Gale, 1985; Halonen et al., 1994; Vismer et al., 2015). Excitatory synaptic function showed only a relatively moderate increase at an intermediate 48-h time point, even using a repeated-injury paradigm. This contrasts with more severe models such as fluid percussion injury and controlled cortical impact where changes last for days to weeks (Santhakumar et al., 2001; Witgen et al., 2005; Cantu et al., 2015). At 1 h after TBI, however, excitatory synaptic input was strongly enhanced and predominated over less pronounced changes in inhibitory input, despite the use of a single-injury rather than a repetitive TBI protocol. Synaptic changes were paralleled by reduced performance in an olfactory discrimination task, where recovery also followed a time course of ∼48 h. Our data suggest that the synaptic consequences of mild TBI evolve rapidly over the hours following injury, highlighting a need to better understand the initial effects of trauma.

### Early Injury Responses

The effects of TBI are often characterized at 24 h or later after injury, reflecting the need to understand chronic pathophysiology. However, a few observations suggest that trauma drives complex changes on more rapid timescales. EEG recordings revealed hyperactivation and brief seizure activity in rat parietal cortex 1–2 min after cortical compression injury, followed by a post-ictal depression lasting ≥2 h (Nilsson et al., 1994). In contrast, *in vivo* Ca^2+^ imaging showed a decrease in hippocampal activity within seconds of blast injury in mice that recovered after ∼60 min (Hansen et al., 2018). Similarly, multi-unit firing in rat neocortex was initially suppressed at 5–15 min after cortical compression, followed by subsequent hyper-responsiveness appearing approximately 2 h after injury (Kharatishvili et al., 2006; Ding et al., 2011). A parallel sequence of suppression followed by hyperexcitability was seen in cortical EEG data after fluid percussion, including epileptic activity emerging hours to weeks later (Kharatishvili et al., 2006). Thus, while trauma responses appear to vary with brain region and injury model, they can also occur within minutes and evolve over time. Our data are consistent with dynamic changes in neural function, showing rapid changes in synaptic efficacy appearing within an hour of injury but largely resolving over the ensuing 48 h. The presence of considerable recovery even with repeated impacts suggests that early time windows should be a major focus in characterizing the effects of mild injury. Given the substantial recovery of synaptic function, we did not make additional measurements at later time points.

The mechanisms that drive initial synaptic changes and subsequent recovery are unclear. In addition to direct mechanical damage, TBI triggers numerous secondary cascades, including mitochondrial dysfunction and elevation of glutamate and intracellular calcium (Povlishock and Katz, 2005; Blennow et al., 2012; Giza and Hovda, 2014). Loss of homeostasis and depolarization can occur within minutes (Blennow et al., 2012; Giza and Hovda, 2014), and may contribute to the changes we observe. Axonal injury may also disrupt synaptic function but is typically thought to occur over hours to days except in cases of severe TBI (Povlishock and Katz, 2005). Recovery may occur through a variety of compensatory mechanisms that adjust excitability and synaptic strength to maintain balanced network activity. A wide range of cellular and synaptic adaptations occur within 24–48 h after perturbation of activity (Turrigiano et al., 1998; Ehlers, 2003; Turrigiano and Nelson, 2004; Keck et al., 2013), and can appear as quickly 1–2 h (Ibata et al., 2008). Structural adaptations such as axonal sprouting and changes in dendritic spines also occur rapidly in response to disruptions such as stroke, injury, or loss of sensory input (Grutzendler et al., 2002; Majewska et al., 2006; Brown et al., 2008; Keck et al., 2011). While long-lasting synaptic changes are common in more severe injury (Santhakumar et al., 2000; Witgen et al., 2005; Kharatishvili et al., 2006; Yang et al., 2010; Alwis et al., 2012; Cantu et al., 2015; Smith et al., 2015), our data suggest that adaptive mechanisms may already be engaged by 48 h after mild injury.

### Injury and Excitatory-Inhibitory Balance

While TBI affected both excitatory and inhibitory inputs, changes in excitation appeared to be more robust, with clear effects on both amplitude and frequency of spontaneous inputs. Loss of E-I balance underlies seizures and epilepsy, which are common outcomes of severe injuries in both humans and animal models (Annegers et al., 1998; Pitkanen et al., 2009). The role of the hippocampus in temporal lobe seizures and memory loss has made it a strong focus of TBI work (Chang and Lowenstein, 2003). Hippocampal function depends on its highly recurrent circuit organization, which is shared by piriform cortex, where pyramidal cells receive thousands of local intracortical connections spanning distances of ≥2mm (Johnson et al., 2000; Franks et al., 2011). Piriform’s highly interconnected excitatory network requires strong inhibition to prevent runaway activity (Johnson et al., 2000; Franks et al., 2011; Bolding and Franks, 2018), and is also strongly linked to seizures and epilepsy. Anterior piriform contains “area tempestas,” a region highly sensitive to chemically induced seizures (Piredda and Gale, 1985; Gale, 1988), which are propagated to other areas by posterior piriform and neighboring regions (Halonen et al., 1994; Tortorella et al., 1997). Seizure-induced hyperactivity and oxidative stress cause localized neuronal loss and atrophy in piriform cortex in both humans and animal models of temporal lobe epilepsy (Candelario-Jalil et al., 2001; Chen and Buckmaster, 2005; Pereira et al., 2005). Despite its involvement in seizure activity in other contexts, piriform has not to our knowledge been examined after TBI.

Multiple mechanisms may underlie early enhancement of excitatory transmission. Increased sEPSC amplitudes could result from strengthening of individual postsynaptic sites via increased receptor content, as with classical activity-dependent plasticity mechanisms (Turrigiano et al., 1998; Ehlers, 2003; Turrigiano and Nelson, 2004; Ibata et al., 2008; Keck et al., 2013). Injury can also increase membrane excitability, however, and changes in levels of resting activity in the piriform network may also play a role. We did not block action potentials in order to measure spontaneous and evoked responses in the same cell, so we cannot clearly distinguish between synaptic input from spontaneous vesicle fusion and release driven by firing in other piriform neurons. Since local connections are typically composed of multiple synaptic contacts (Franks and Isaacson, 2006), elevated spontaneous activity would also generate additional larger events. Similarly, changes in inhibitory frequency may reflect changes in either presynaptic release probability, spontaneous firing, or both.

Given the bias toward enhancement of spontaneous excitatory input, the lack of significant changes in evoked E-I balance was unexpected. We note that we did find a strong trend toward increased excitation at 1 h after TBI (*p* = 0.082), as well as high variability consistent with heterogeneous injury effects in both animal models and humans (Carroll et al., 2004; Lingsma et al., 2010; Xiong et al., 2013). Another possibility is that TBI acts preferentially on intracortical associational synapses rather than ascending afferent inputs. Intracortical inputs will be emphasized in spontaneous measurements as their more proximal dendritic location will be less affected by electrotonic filtering (Bathellier et al., 2009). LOT stimulation primarily drives afferent inputs onto distal dendrites, followed by a smaller secondary input from intracortical circuits. Determining the cause of the E-I imbalance is an important question for future studies. Pharmacological methods may help test for selective changes in ascending pathways versus local intracortical circuits (Tang and Hasselmo, 1994; Franks and Isaacson, 2005).

### Involvement of Piriform in Injury

We consistently found neuroinflammation in piriform and surrounding areas, which have previously received little attention in TBI research. While trauma-induced damage has been described in diverse brain areas, including somatosensory and prefrontal cortex, the majority of work has focused on hippocampus (Reeves et al., 1995, 1997; Santhakumar et al., 2000, 2001; Golarai et al., 2001; Zanier et al., 2003; Witgen et al., 2005; Griesemer and Mautes, 2007; Hansen et al., 2018). While we did find mild hippocampal neuropathology in one case, it was inconsistent across animals. The reasons for selective damage in piriform and entorhinal areas are unclear but may be due to the free head movement allowed by our model. Activated microglia were most prominent in ventrolateral areas opposite the dorsal impact site, consistent with a coup-contrecoup effect causing damage on the opposite side of the brain (Courville, 1942). Piriform’s extensive axonal pathways may also make it particularly sensitive to diffuse axonal injury, which tends to affect long-range projections (Pandit et al., 2013; Caeyenberghs et al., 2014). Afferent inputs to piriform course ≥5 mm along the base of the brain, and both intracortical fibers and piriform projections extend over longer distances than those in neocortex (Johnson et al., 2000; Franks et al., 2011).

Disruptions in piriform are consistent with a range of sensory deficits described in both human injury and animal models, including visual and auditory systems as well as olfaction (Ciuffreda et al., 2007; Lew et al., 2007a; Alwis et al., 2012; Proskynitopoulos et al., 2016). Reduced odor discrimination ability is consistent with the relatively localized microglial activation we found in piriform. Interestingly, changes in synaptic function and discrimination ability shared broadly similar time courses, being most severe at earlier time windows and largely recovering by approximately 48 h. As we used a diffuse injury model, and did not record from other brain regions, changes in other areas may also contribute to loss of discrimination ability. Further work will be needed to test whether synaptic disruptions are confined to piriform or extend more widely across brain areas. More generally, sensory assays have potential as a simple and quantitative way to assess TBI severity, both during early post-injury phases as well as longer-term effects (Bressler et al., 2017).

### Outlook

The fast onset and subsequent recovery of synaptic changes is a key finding of our study, highlighting the need to address the complex interactions between initial damage and compensatory responses during early phases of TBI. Long-term pathology and/or neurodegeneration are initiated by processes occurring at the time of injury (McKee et al., 2009; Goldstein et al., 2012), and a deeper understanding of early time windows will be critical for targeting appropriate interventions to different injury phases. While our *in vitro* approach allowed detailed synaptic characterization, it may not reveal all of the factors contributing to injury in the intact brain, potentially accounting for why vestibulomotor deficits persisted at 48 h even though synaptic changes had largely recovered. Slicing removes a large portion of network interactions and substantially reduces background activity (Lipton and Whittingham, 1984; Moyer and Brown, 2002; Hájos et al., 2009), potentially countering the effects of hyperexcitability. TBI also dramatically reduces cerebral blood flow (Golding, 2002; DeWitt and Prough, 2003; Kenney et al., 2016), compounding trauma with metabolic stress. Slices are perfused with oxygenated recording solutions that could alleviate these energetic factors. Interestingly, the increases in excitatory input we see here have parallels with synaptic responses in hypoxia, stroke, and epilepsy (Fleidervish et al., 2001; Bolay et al., 2002; Hofmeijer and Putten, 2012; Naylor et al., 2013). It will be important for future work to examine how mild injury acts *in vivo* to better capture the interactions between synaptic disruptions, altered excitability, and oxidative stress.

## Ethics Statement

This study was performed in strict accordance with the recommendations in the Guide for the Care and Use of Laboratory Animals of the National Institutes of Health. All animals were handled according to approved Institutional Animal Care and Use Committee (IACUC) protocols (#17-017) of Boston University.

## Author Contributions

EW, YG, AG, IM, AM, and ID designed and performed the experiments. EW, GD, WE, and ID analyzed the data. EW, GD, WE, and ID wrote the manuscript.

## Conflict of Interest Statement

YG is currently an employee of Akuous, Inc. The remaining authors declare that the research was conducted in the absence of any commercial or financial relationships that could be construed as a potential conflict of interest.

## Footnotes

1 https:sites.google.com/site/tarotoolsmember/
